# Mechanisms and Applications of the Anti-cancer Effect of Pharmacological Ascorbic Acid in Cervical Cancer Cells

**DOI:** 10.3389/fonc.2020.01483

**Published:** 2020-09-02

**Authors:** Tsai-Ming Wu, Shu-Ting Liu, Ssu-Yu Chen, Gunng-Shinng Chen, Chia-Chun Wu, Shih-Ming Huang

**Affiliations:** ^1^Department of Biochemistry, National Defense Medical Center, Taipei City, Taiwan; ^2^Department of Dentistry of Tri-service General Hospital, School of Dentistry, National Defense Medical Center, Taipei City, Taiwan; ^3^Department of Orthopaedic Surgery, Tri-service General Hospital, National Defense Medical Center, Taipei City, Taiwan

**Keywords:** L-ascorbic acid, p53, ER stress, reactive oxygen species, Nrf2

## Abstract

In recent years, L-ascorbic acid (L-AA), or vitamin C, has been attracting attention as a potential anticancer drug that mediates hydrogen peroxide-induced oxidation and ten-eleven translocation 2-catalyzed DNA demethylation. However, the precise mechanism by which L-AA acts remains unclear. We examined the cytotoxic effects of L-AA or sodium ascorbate in human cervical carcinoma cells by assessing cell viability, expression of cell cycle-related mRNAs and proteins, and mitochondrial functions, and by performing flow cytometric analyses of cell cycle profiles, apoptosis, cell proliferation, and production of reactive oxygen species (ROS). We later tested the effects of ascorbates in combination with two first-line chemotherapeutic drugs, cisplatin, and doxorubicin. At pharmacological concentrations (1–10 mM), L-AA increased ROS levels; decreased levels of several cell cycle-related proteins, including p53, p21, cyclin D1, and phosphorylated histone 3 at serine residue 10; induced DNA damage, as indicated by changes in γH2A.x; decreased levels of the anti-oxidative transcription factor Nrf2; and increased levels of catalase, superoxide dismutase 1, and endoplasmic reticulum stress-related indicators, such as the p-eIF2α/eIF2α ratio and CHOP levels. L-AA also promoted cell proliferation and induced apoptosis and mitochondrial dysfunction. Finally, L-AA increased the susceptibility of HeLa cells to cisplatin and doxorubicin. These findings provide insight into how the adjustment of the cellular ROS status through L-ascorbate (L-AA or sodium ascorbate) administration could potentially synergistically enhance the efficacy of cancer therapies.

## Introduction

L-ascorbic acid (L-AA), also known as vitamin C, is an essential nutrient found in a variety of fruits and vegetables and is also available as a dietary supplement (1). L-AA can undergo two consecutive single-electron oxidations by which result in the formation of dehydroascorbic acid. Moreover, this oxidation is enhanced in the presence of a catalytic metal, enabling L-AA to promote oxidation via hydrogen peroxide generation through pH-dependent auto-oxidation (2). Ascorbate functions as a cofactor for the reduction of various enzymes, including hydroxylases in the Fe^2+^-2-oxoglutarate-dependent dioxygenase family (3). In part, these enzymes contribute to the stability of hypoxia-inducible factor 1 alpha (HIF-1α) and Tet2 (ten-eleven translocation 2) DNA hydroxylases, which catalyze the demethylation of epigenetically modified DNA (4–7). L-AA may also be involved in tissue repair and several other coenzyme functions; however, details of the working mechanisms remain unclear.

Epidemiological evidence suggests that foods rich in L-AA can play a protective role against tumorigenesis. For example, plasma L-AA concentrations are reported to be inversely associated with cancer risk (8–10), though large-scale randomized interventions with L-AA alone or in combination with other antioxidants (vitamins A, C, E, and beta-carotene) showed no protection (11). The control of L-AA absorption from the intestine is very strict, but recent pharmacokinetic data suggest that intravenous L-AA can bypass this tight control leading to higher plasma levels (12, 13). As a result, L-AA has been redefined as a potential anticancer drug in recent years (4, 6, 14).

Oxidative stress results from an imbalance between the production of reactive oxidative species (ROS) and antioxidant capacity (15). ROS include superoxide anion, hydroxyl radical, hydrogen peroxide, and singlet oxygen, while the antioxidant enzymes include superoxide dismutase (SOD), catalase, glutathione peroxidase, and glutathione-S-transferase. Oxidative damage is not only a cause but also a consequence of various types of cell death. As a potential cancer treatment strategy, elevated oxidative stress within cancer cells achieved through the inhibition of antioxidant enzymes or production of ROS can lead to lipid peroxidation and damage to proteins and DNA (16–18). For example, the hydrogen peroxide formed at pharmacological L-AA concentrations exhibits potential therapeutic efficacy against cancer, acting both intracellularly and extracellularly (19–22). This suggests that L-AA at pharmacological concentrations may serve as an anti-cancer drug. However, it is not well-clear why L-AA kills some cancer cells through mechanisms related to hydrogen peroxide but has little effect on normal cells (23).

Due to fundamental defects in oxidative metabolism and an increased pool of labile iron, the cancer cells have generally been hypothesized to demonstrate higher steady-state levels of ROS stress than normal cells. In the present study, we examined the anti-oxidative and oxidative actions of L-ascorbates (L-AA or sodium ascorbate) within cancer cells at physiological and pharmacological concentrations. In addition to its effects on ROS, our study also addressed the functional impact of pharmacological concentrations of L-AA on cell cycle profiles, antioxidant response, mitochondrial dysfunction, and endoplasmic reticulum (ER) stress. We hope that our findings will open a new direction focusing on the adjustment of cellular ROS via L-ascorbates (L-AA or sodium ascorbate) for cancer therapy.

## Materials and Methods

### Cell Culture and Chemicals

HeLa cervical carcinoma (ID 60005), C33A (ID 60554), and SiHa (ID 60528) cells were purchased from Bioresource Collection and Research Center, Taiwan, Republic of China. Three cervical carcinoma cells were cultured in Dulbecco's modified Eagle's medium (DMEM) containing 10% fetal bovine serum (FBS) and 1% penicillin-streptomycin (Thermo Fisher Scientific, CA, USA). L-AA (PubChem CID:54670067), acetic acid (PubChem CID:176), sodium ascorbate (PubChem CID:23667548), cisplatin (PubChem CID:441203), and doxorubicin hydrochloride (PubChem CID:443939) were all from Sigma Aldrich (MO, USA).

### Cell Survival Analysis

Cells (5 × 10^4^ cells/well) were plated in 24-well culture plates and cultured for about 24 h. Then, the cells were treated with various concentrations of L-AA in fresh DMEM for the indicated times. The interference of L-AA with the MTT assay had been reported (24). Therefore, before adding the MTT solution (0.5 mg/ml in phosphate-buffered saline, PBS) to each well, the medium containing L-ascorbic acid must be removed, and the cells are washed twice by PBS to avoid the interaction between L-AA and MTT. The cells were then incubated with the MTT solution for 1 h at 37°C. Dimethyl sulfoxide (DMSO; 200 μl) was then added, and the absorbances at 570 nm and 650 nm were measured using an ELISA plate reader (Multiskan EX, Thermo, MA, USA). The control group containing cells cultured in medium only was defined as 100% cell survival. Combination index (CI) was calculated using CalcuSyn (Biosoft, Cambridge, UK) to generate the isobolograms. Generally, CI <1 indicates a synergistic combination effect and CI >1 indicates an additive combination effect (25).

### Fluorescence-Activated Cell Sorting (FACS), Cell Cycle Profiles, Apoptosis, ROS, and Proliferation Analysis

At the end of treatment, cells were harvested and fixed in 70% ice-cold ethanol. After being stored at −30°C overnight, the fixed cells were washed twice using ice-cold PBS containing 1% FBS and stained with a propidium iodide (PI) solution (5 μg/ml PI in PBS, 0.5% Triton x-100, and 0.5 μg/ml RNase A) for 30 min at 37°C in the dark. According to cellular DNA content, the cell cycle distribution was measured by FACS. All samples were analyzed using the FACSCalibur flow cytometer (BD Biosciences, CA, USA). Data were analyzed using the Cell Quest Pro software (BD Biosciences).

Early and late stages of apoptotic cells were evaluated by a fluorescein Phycoerythrin (PE)-Annexin V Apoptosis Detection Kit (BD Biosciences) according to the manufacturer's protocol. Cells were stained with PE-Annexin V as well as 7-Amino-Actinomycin (7-AAD) to allow the investigator to identify early apoptotic cells. Viable cells were PE Annexin V and 7-AAD negative; early apoptotic cells were PE Annexin V positive and 7-AAD negative; and late apoptotic or dead cells were both PE Annexin V and 7-AAD positive.

The levels of intracellular ROS were measured by fluorescent marker DCFH-DA. Cells (2 × 10^5^ cells/well) were treated with various concentrations of L-AA for 24 h, after which they were incubated with DCFH-DA (10 μM) for 40 min at 37°C in the dark. After being harvested and washed with PBS, the cells were evaluated using a FACSCalibur flow cytometer and Cell Quest Pro software.

For proliferation analysis, the cells (2 × 10^5^ cells/well) were treated and processed with FITC-BrdU (5-bromo-2-deoxyuridine) Flow Kits according to the manufacturer's instructions (BD Biosciences). All samples were analyzed using the FACSCalibur flow cytometer. Data were analyzed using the Cell Quest Pro software.

### Western Blot Analysis

HeLa, SiHa, and C33A cells (2 × 10^5^ cells/well) were lysed using RIPA buffer (100 mM Tris-HCl (pH 8.0), 150 mM NaCl, 0.1% SDS, and 1% Triton 100) at 4°C. The concentration of proteins in the resultant lysate were evaluated by a DC Protein Assay Kit (Bio-Rad Laboratories, CA, USA), after which they were separated by SDS-PAGE and then electrotransferred to PVDF membranes (Immobilon-P; Millipore, Bedford, MA, USA) using a Bio-Rad Semi-dry Transfer Cell. The blots were then incubated with primary antibodies against α-actinin (ACTN) (SC-17829), catalase (SC-271803), cyclin B1 (SC-245), H3P (SC-8656-R), HuR (SC-5261), Nrf2 (SC-365949), SOD1 (SC-101523), and p53 (SC-126) (Santa Cruz Biotechnology, CA, USA); CHOP (#2895), EGFR (#4267), eIF2α (#9722), p-eIF2α (#9721), and LC3B (#2775) (Cell Signaling, MA, USA); and cyclin D1 (ab134175), γH2A.x (ab81299), p21 (ab109520), and survivin (ab76424) (Abcam, Cambridge, UK), and HO-1 (ADI-SPA-895-F) (Enzo Life Sciences, NY, USA). Next, the blots were incubated with horseradish peroxidase-conjugated secondary antibody (Santa Cruz Biotechnology). The immunoreactive proteins were then detected using ECL™ Western Blotting Detection Reagents and Amersham Hyperfilm™ ECL (GE Healthcare, TX, USA).

### Reverse Transcriptase Polymerase Chain Reaction (RT-PCR)

HeLa cells (2 × 10^5^ cells/well) were lysed by TRIzol reagent (Invitrogen) to isolate total RNAs. Reverse transcription for first-strand cDNA synthesis was conducted using MMLV reverse transcriptase (Epicenter Biotechnologies, WI, USA) with 1 μg of total RNA for 60 min at 37°C. PCR reactions were operated on a Veriti Thermal Cycler (Applied Biosystems, MA, USA). The PCR primers are listed in Table 1.

**Table 1:** PCR primers used in this study.

**Gene name**	**Primer sequence (5′ → 3′)**
*p53*	Forward: 5′-CTCTGACTGTACCACCATCCACTA-3′ Reverse: 3′-GAGTTCCAAGGCCTCATTCAGCTC-3′
*p21*	Forward: 5′-CTGAGCCGCGACTGTGATGCG-3′ Reverse: 5′-GGTCTGCCGCCGTTTTCGACC-3′
*Cyclin D1*	Forward: 5′-ATGGAACACCAGCTCCTGTGCTGC-3′ Reverse: 5′-TCAGATGTCCACGTCCCGCACGTCGG-3′
*Survivin*	Forward: 5′-ATGGGTGCCCCGACGTTG-3′ Reverse: 5′-AGAGGCCTCAATCCATGG-3′
*Cyclin B1*	Forward: 5′-GTTGATACTGCCTCTCCAAG-3′ Reverse: 5′-CTTAGTATAAGTGTTGTCAGTCAC-3′
*Nrf2*	Forward: 5′-CAGTCAGCGACGGAAAGAGT-3′ Reverse: 5′-GGCTACCTGAGCAACAGAAG-3′
*HO-1*	Forward: 5′-ATGCCCCAGGATTTGTCAGAG-3′ Reverse: 5′-AGGGCTTTCTGGGCAATCTTT-3′
*p62*	Forward: 5′-CCGTGAAGGCCTACCTTCTG-3′ Reverse: 5′-GCACTTGTAGCGGGTTCCTA-3′
*GAPDH*	Forward: 5′-CTTCATTGACCTCAACTAC-3′ Reverse: 5′-GCCATCCACAGTCTTCTG-3′

### Whole Cell, Cytoplasmic Extract, and Nuclear Extract Preparations

HeLa cells (2 × 10^5^ cells/well) were seeded in 100-mm culture dishes, cultured until they reached about 80–90% confluence, and harvested. Cells were lysed in RIPA buffer at 4°C to prepare whole cell extract, while soluble extracts were obtained by centrifugation (14,000 × *g*, 15 min, 4°C). The cytoplasmic and nuclear extracts were prepared with a ProteoExtract^®^ Subcellular Proteome Extraction Kit (Merck Millipore, MA, USA).

### Transient Transfection and Measurement of Relative Luciferase Activity

Reporter NRF2(ARE)-LUC was purchased from the Signosis Company (Cat# LR-2106, CA, USA). HeLa cells (5 × 10^4^ cells/well) were plated in 24-well plates, and 1.0 μg of reporter were transfected using jetPEI (Polyplus Transfection Inc., NY, USA) following the manufacturer's protocol (Promega Luciferase Assay Kit and DLR2 model). Cells were harvested for luciferase reporter assays using a Promega Luciferase Assay Kit as described previously (26). Luciferase activities in extracts from the transfected cells are presented in terms of relative light units (RLU) and expressed as the mean and standard deviation of three transfected cultures.

### Detection of Oxygen Consumption (OCR) and Extracellular Acidification (ECAR)

Cellular OCR and ECAR were measured using a Seahorse XF24 bioenergetic assay according to the manufacturer's instructions (Agilent, CA, USA). Procedural details were as previously described (27, 28). Briefly, HeLa cells (5 × 10^3^ cells/well) were plated on an XF24 microplate and cultured for 3 days. Thereafter, XF24 bioenergetic assays were started by replacing the exhausted medium with sodium bicarbonate-free DMEM (pH 7.4) supplemented with 2% FBS and 2% horse serum. The OCR and ECAR were measured at a steady state, and then oligomycin (1 μM), carbonyl cyanide 4-[trifluoromethoxy] phenylhydrazone (FCCP; 0.5 μM), and rotenone/antimycin (0.5 μM) were added sequentially into the wells to obtain values for the maximal and non-mitochondrial respiration rates.

### Statistical Analysis

Values are expressed as the mean ± SD of at least three independent experiments. All comparisons between groups were made using Student's *t*-tests. Comparison among multiple groups was conducted using analysis of variance (ANOVA). Statistical significance was set at *p* < 0.05.

## Results

### Effects of L-AA on Cell Survival, Cell Cycle Profiles, Apoptosis, and Cellular Proliferation in HeLa Cells

**Figure 1:**
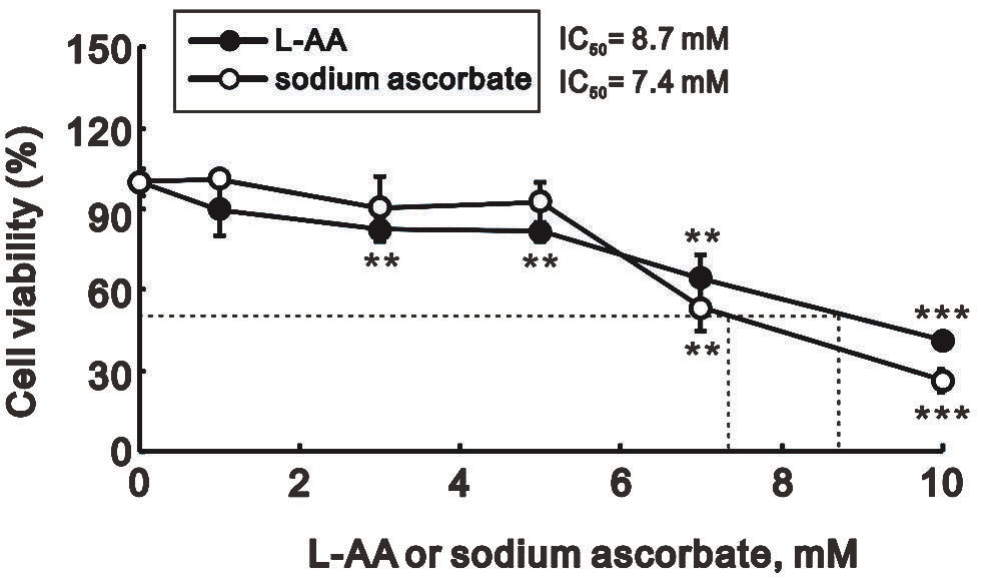
Cytotoxicity of L-AA in HeLa cells. HeLa cells (5 × 10^4^ cells/well) were treated with the indicated concentrations of L-AA or sodium ascorbate for 24 h. Cell viability was measured using the MTT method. The results are representative of three independent experiments. ^**^p < 0.01 and ^***^p < 0.001 (Student's *t*-tests).

**Figure 2:**
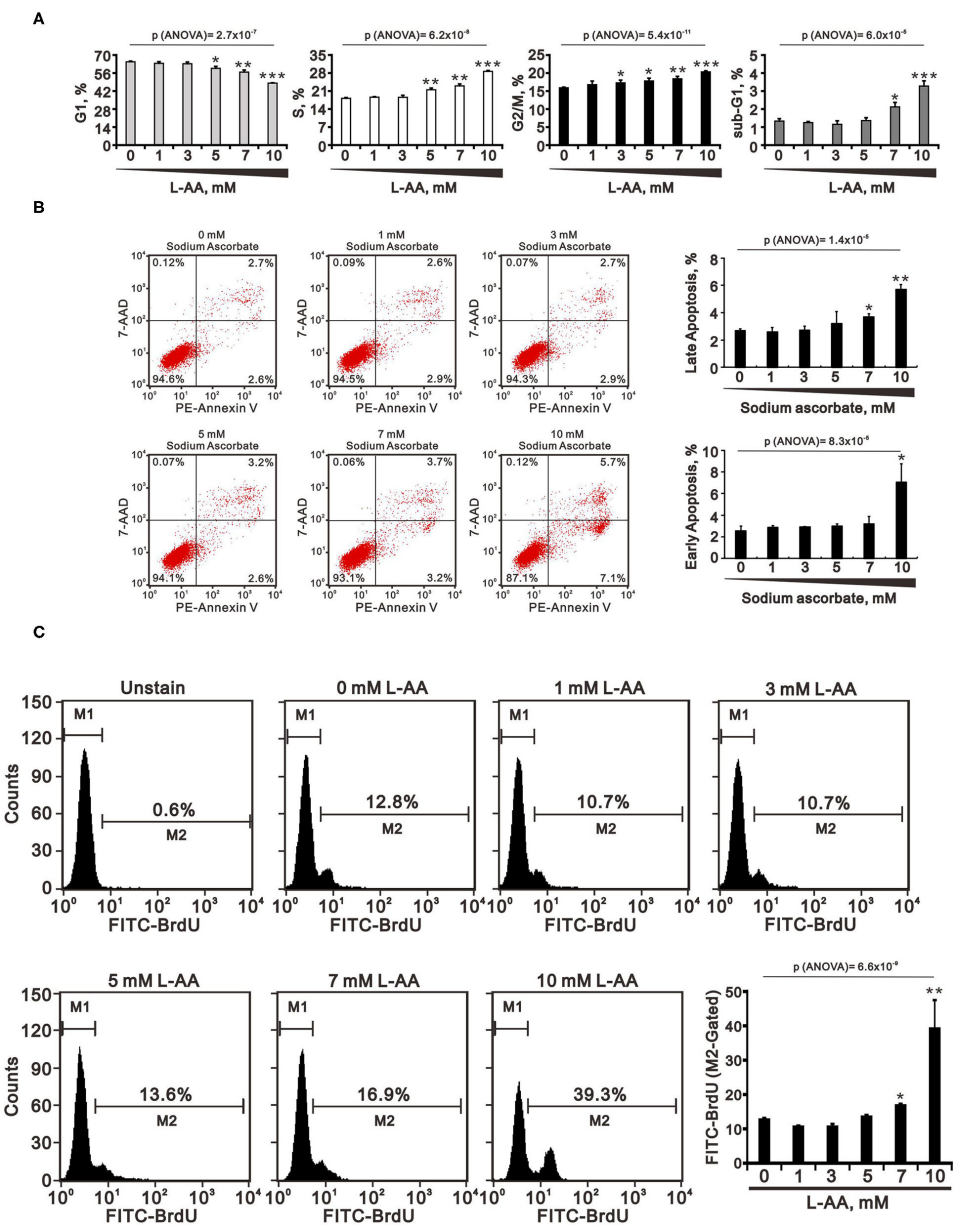
Effects of L-AA or sodium ascorbate on the cell cycle profiles, apoptosis, and cellular proliferation in HeLa cells. **(A**–**C)** HeLa cells (2 × 10^5^ cells/well) were incubated for 40 h with the indicated concentration of L-AA or sodium ascorbate. They were then subjected to flow cytometric cell cycle profiling **(A)**, annexin V staining **(B)**, and BrdU proliferation analysis **(C)**. Early apoptotic cells are PE Annexin V positive and 7-AAD negative; and late apoptotic cells are both PE Annexin V and 7-AAD positive. The results are representative of three independent experiments. ^*^p < 0.05, ^**^p < 0.01, and ^***^p < 0.001 (Student's *t*-tests).

**Figure 3:**
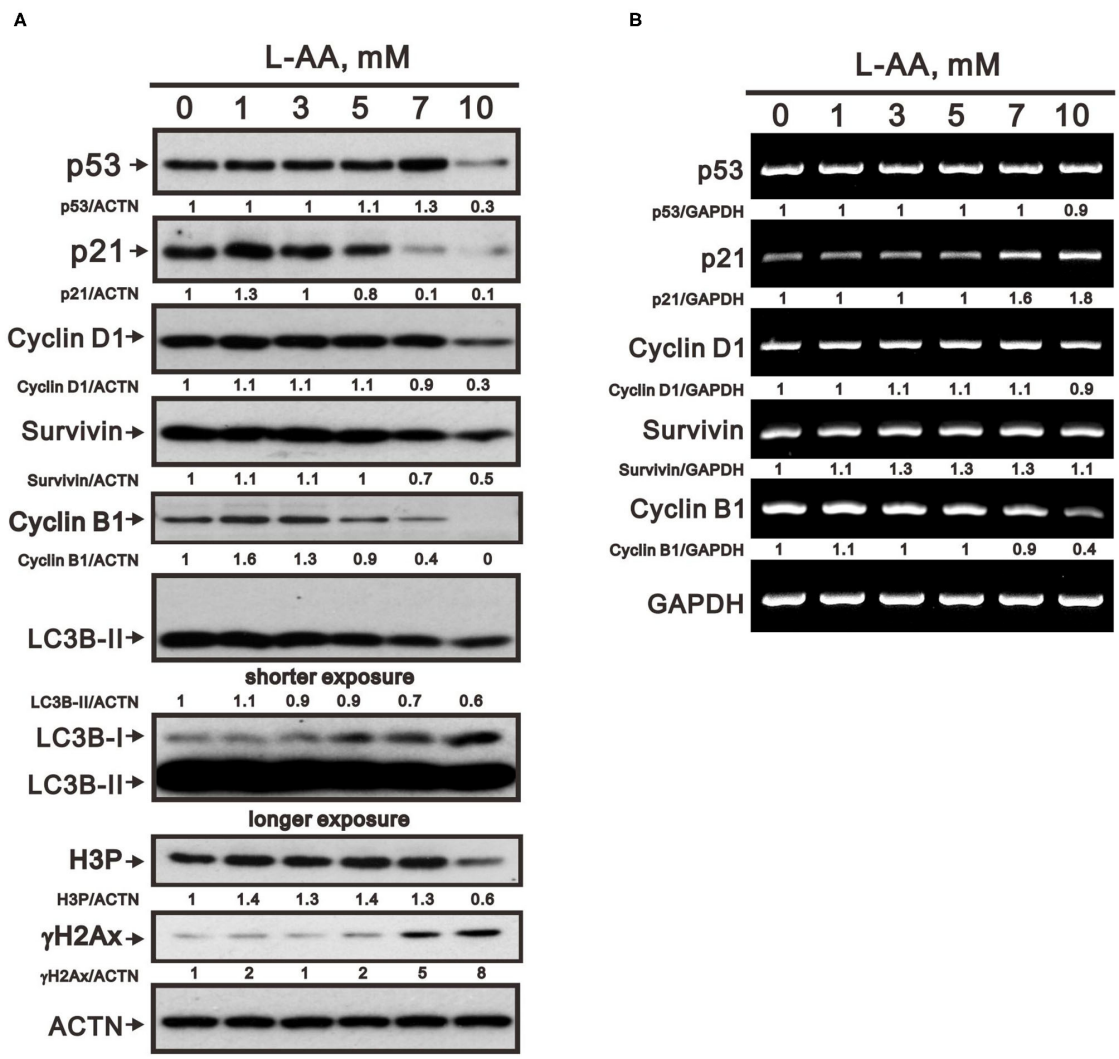
Effects of L-AA on expression of cell cycle-related proteins in HeLa cells. **(A,B)** HeLa cells (2 × 10^5^ cells/well) were incubated for 40 h with the indicated concentrations of L-AA and then lysed. The cell lysates were subjected to Western blot analysis using antibodies against p53, p21, cyclin D1, Survivin, cyclin B1, LC3B, H3P, and γH2A.x **(A)**; or to RT-PCR analysis for *p53, p21, cyclin D1, Survivin*, and *cyclin B1* **(B)**. ACTN was the protein loading control; *GAPDH* mRNA was the mRNA loading control. The results **(A,B)** are representative of three independent experiments. Protein and PCR bands were quantified through pixel density scanning and evaluated using ImageJ, version 1.44a (http://imagej.nih.gov/ij/). The fold was normalized to the internal control protein (ACTN) or gene (GAPDH).

The actions of L-AA are dependent on its concentration. Here, we sought to examine the effects of pharmacological concentrations (>1 mM) of L-ascorbates (L-AA and sodium ascorbate) in HeLa cervical carcinoma cells. Using MTT assays, we first determined that IC_50_ values for L-AA and sodium ascorbate were about 8.7 and 7.4 mM, respectively, at a 24-h time point (Figure 1). We then assessed the effect of pharmacological concentrations of L-AA on the cell cycle profile, apoptosis, and proliferation in HeLa cells. In a flow cytometric analysis, we observed that the subG1, S, and G2/M populations were all dose-dependently increased, while the G1 population was decreased (Figure 2A). In the annexin V staining analysis, we found significant increases in the percentage of early apoptotic cells at 10 mM sodium ascorbate (Figure 2B), which were consistent with the subG1 population in Figure 2A. Using BrdU proliferation assays, the highest M2 phase fraction was observed at 10 mM L-AA, suggesting that cellular proliferation is promoted by L-AA excess 7 mM (Figure 2C). In Western blotting and RT-PCR analyses, we examined the proteins related to apoptosis (p53 and survivin), cell cycle (p53, p21, cyclin D1, cyclin B1, and H3P), survival (survivin), autophagy (LC3BII), and DNA damage (γH2A.x). Our results showed that protein levels of p53, p21, cyclin D1, survivin, LC3BII, cyclin B1, and H3P were dose-dependently decreased, whereas protein levels of the DNA damage biomarker γH2A.x were increased (Figure 3A). On the other hand, although mRNA levels of *cyclin B1* were decreased, mRNA levels of *p53, cyclin D1*, and *survivin* remained constant, and mRNA levels of *p21* were increased at higher L-AA concentrations (Figure 3B).

### Effects of L-AA on the Production of ROS and Anti-oxidative Proteins in HeLa Cells

**Figure 4:**
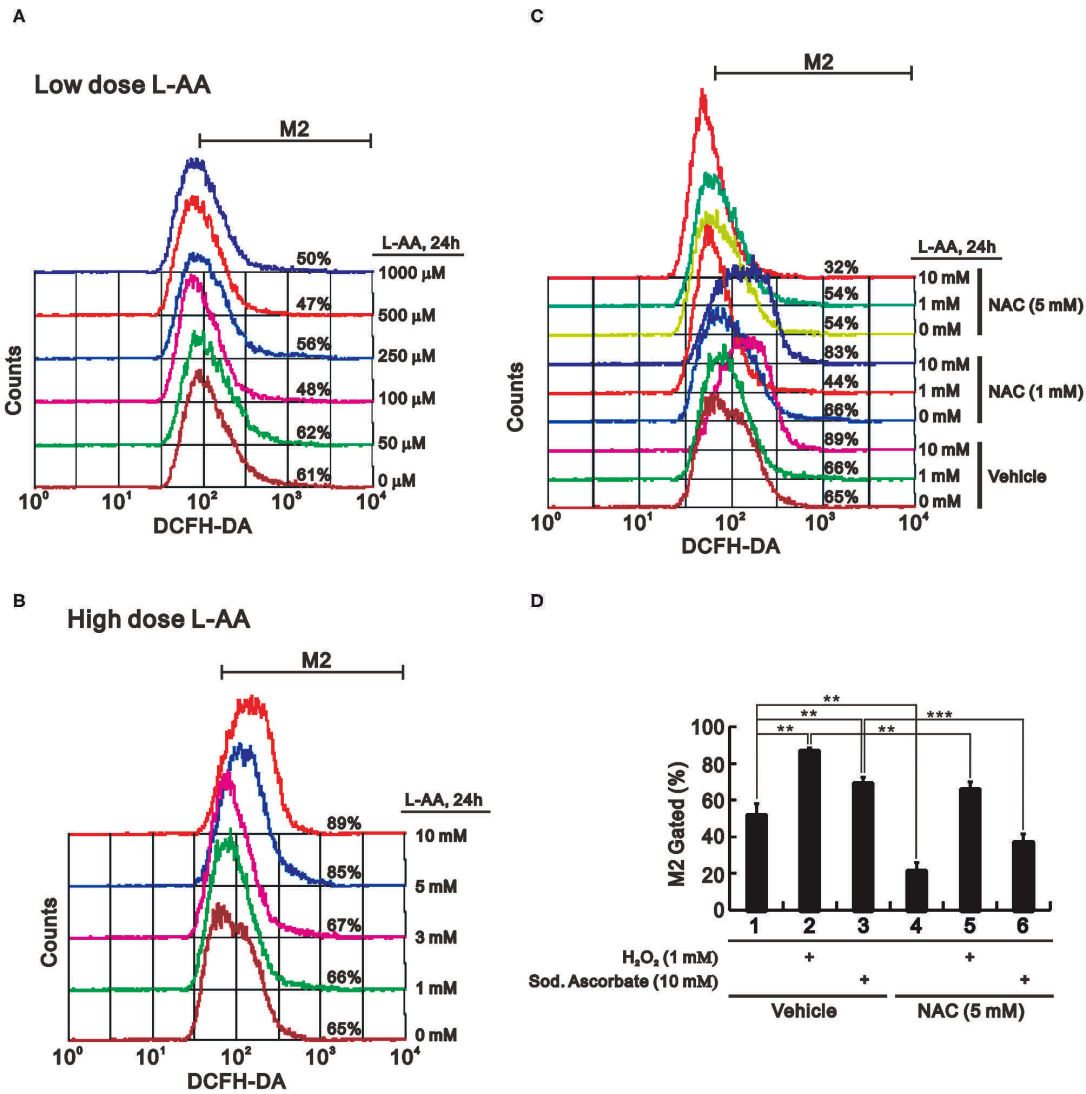
Effect of L-AA on ROS levels in HeLa cells. **(A,B)** HeLa cells (2 × 10^5^ cells/well) were incubated for 24 h with low **(A)** or high **(B)** concentrations of L-AA, the indicated concentration of L-AA plus 1 mM or 5 mM NAC **(C)**, or 1 mM hydrogen peroxide and 5 mM sodium ascorbate combined with 5 mM NAC **(D)**. The live cells were then stained with 10 μM DCFH-DA and assayed using a flow cytometer. The results are representative of three independent experiments. ^**^p < 0.01 and ^***^p < 0.001 (Student's *t*-tests).

To determine the effects of physiological and pharmacological concentrations of L-AA on oxidative stress, we used flow cytometry to measure ROS levels. At physiological concentrations (below 1 mM), we observed a dose-dependent leftward trend in the DCFH-DA signal and a corresponding decrease in the M2 population (Figure 4A). At pharmacological concentrations (from 1 to 10 mM), we observed a dose-dependent rightward shift in the DCFH-DA signal and a corresponding increase in the M2 population (Figure 4B). This suggests that L-AA may act as an antioxidant at lower concentrations, while it can act as an oxidant at higher concentrations. Moreover, the ROS scavenger N-acetylcysteine (NAC) at 1 mM selectively suppressed ROS production induced by 1 mM L-AA, and 5 mM NAC suppressed the ROS induced by 1 mM or 10 mM L-AA (Figure 4C). Background levels of ROS were suppressed by 5 mM NAC, not by 1 mM NAC, under our experimental conditions. Like hydrogen peroxide, sodium ascorbate increased levels of cytosolic ROS, and that effect was significantly suppressed by NAC (Figure 4D).

**Figure 5:**
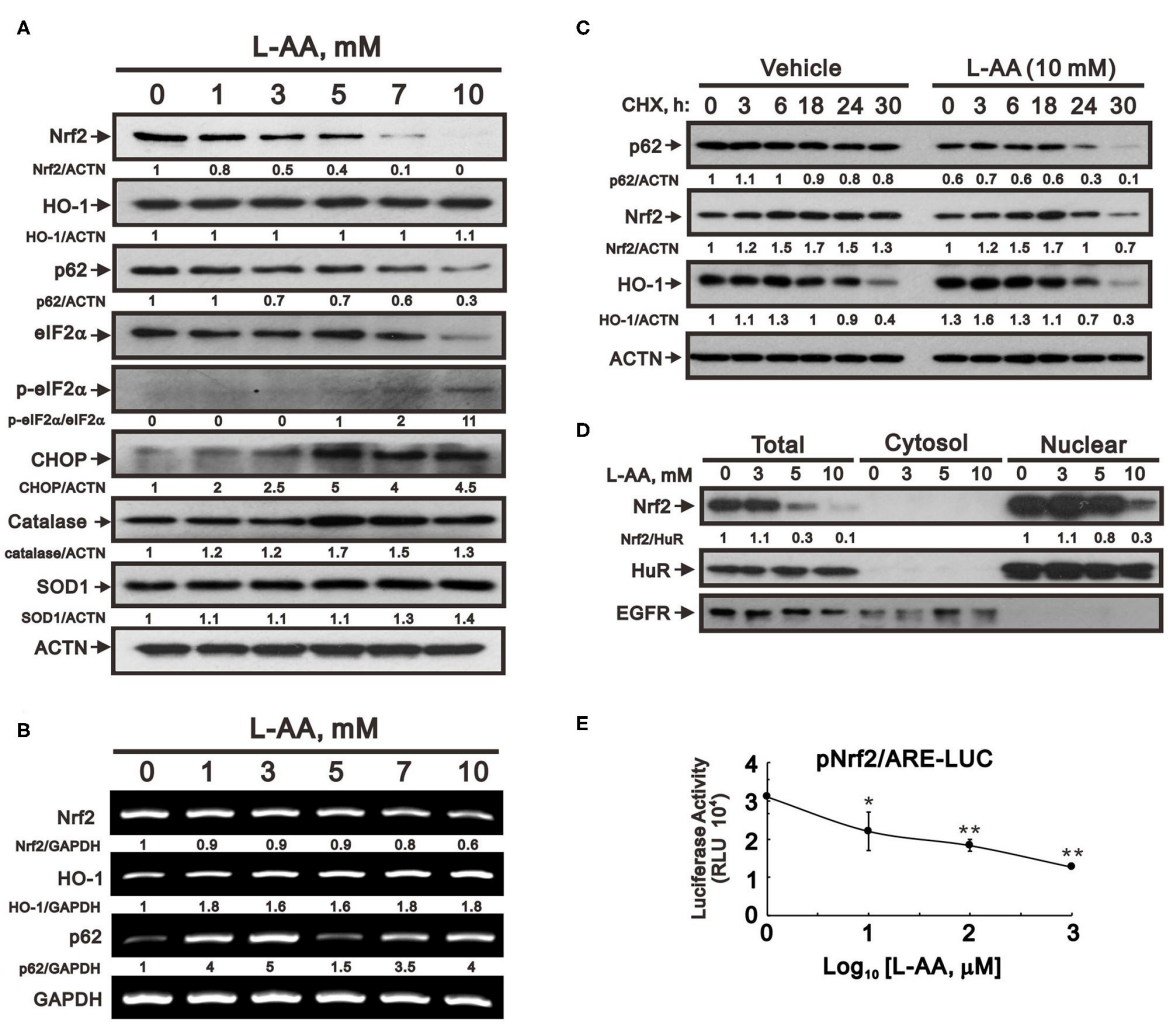
Effect of L-AA on expression of antioxidant proteins in HeLa cells. **(A,B)** HeLa cells (2 × 10^5^ cells/well) were incubated for 24 h with the indicated concentration of L-AA and then lysed. The cell lysates were subjected to Western blot analysis using antibodies against Nrf2, HO-1, p62, e-IF2α, p-e-IF2α, CHOP, catalase, and SOD1 **(A)**; or to RT-PCR analysis for *Nrf2, HO-1*, and *p62* **(B)**. ACTN was the protein loading control; *GAPDH* mRNA was the mRNA loading control. **(C)** HeLa cells (2 × 10^5^ cells/well) were incubated with or without 10 mM L-AA plus 1 μg/ml CHX for the indicated times. Cell lysates were then subjected to Western blot analysis using antibodies against p62, Nrf2, and HO-1. ACTN served as the loading control. **(D)** HeLa cells (2 × 10^5^ cells/well) were incubated for 24 h with the indicated concentrations of L-AA. Fractionated cell lysates were subjected to Western blot analysis using antibodies against Nrf2, HuR (as a marker of the nuclear fraction), and EGFR (as a marker of the cytosolic fraction). The results **(A–D)** are representative of three independent experiments. Protein and PCR bands were quantified through pixel density scanning and evaluated using ImageJ, version 1.44a (http://imagej.nih.gov/ij/). The fold was normalized to the internal control protein (ACTN or HuR) or gene (GAPDH). **(E)** HeLa cells (5 × 10^4^ cells/well) were transiently transfected with 0.5 μg of *Nrf2/ARE-LUC* reporter and treated with the indicated concentration of L-AA for 24 h. The results are representative of three independent experiments. ^*^p < 0.05 and ^**^p < 0.01 (Student's *t*-tests).

Given that pharmacological concentrations of L-AA induced ROS production, we tested whether it would induce Nrf2, a key transcriptional factor in the antioxidant scavenger system. Unexpectantly, levels of both Nrf2 protein and mRNA were dose-dependently decreased by L-AA in HeLa cells (Figures 5A,B). Levels of heme oxygenase 1 (HO-1) protein, a well-known target of Nrf2, were unaffected by L-AA, but HO-1 mRNA was induced by L-AA in a dose-dependent manner (Figures 5A,B). p62 protein, another Nrf2 target, was consistently decreased by L-AA, while changes in p62 mRNA depended on the dose (Figures 5A,B). An examination of two well-known antioxidant enzymes, catalase and SOD1, revealed that levels of both proteins were increased at higher L-AA concentrations (Figure 5A). ROS-mediated ER stress has been extensively studied (29, 30). We observed that ER stress, as reflected by the p-eIF2α/eIF2α ratio and levels of its downstream mediator CHOP, were increased at higher L-AA concentrations (Figure 5A). To assess the stability of Nrf2, p62, and HO-1 proteins in the presence of L-AA, we measured their levels after treating HeLa cells with a de novo protein synthesis inhibitor, cycloheximide (CHX). Our results suggest L-AA may decrease the stability of Nrf2 and p62, but not HO-1 proteins (Figure 5C). Since Nrf2 is a well-known transcription factor, we next examined the nuclear amounts and transcriptional function of Nrf2. Cell fractionation revealed that nuclear Nrf2 levels were predominantly decreased by L-AA (Figure 5D). We also found that L-AA suppressed Nrf2 responsive element reporter activity (Figure 5E).

### Effects of L-AA on Nrf2, p62, and the p-eIF2α/eIF2α Ratio Are Observed in Other Human Cervical Cancer Cells and With Sodium Ascorbate Salt

**Figure 6:**
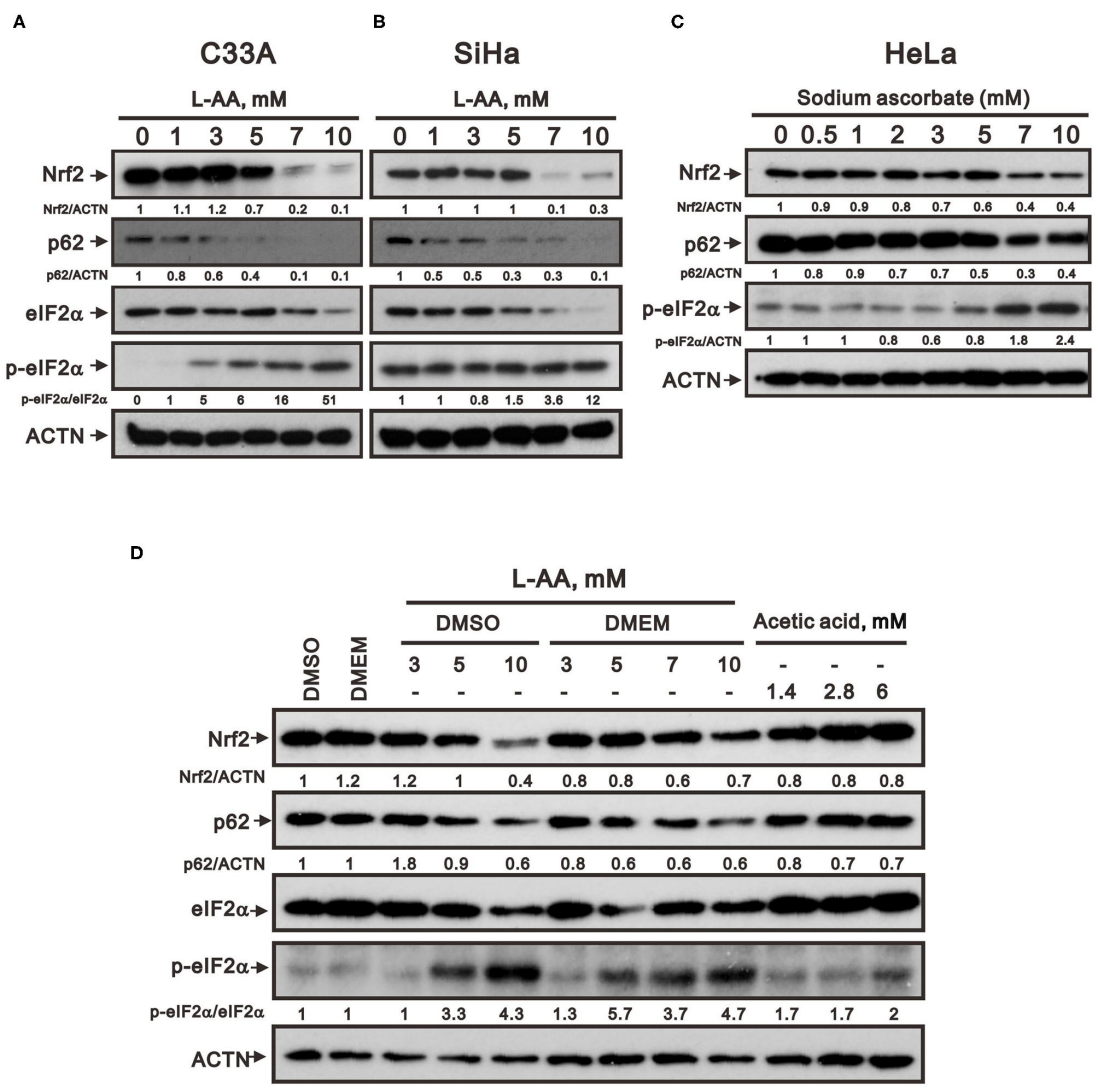
Effects of L-AA on other human cervical cancer cell lines. C33A **(A)** and SiHa **(B)** cells (2 × 10^5^ cells/well) were incubated for 24 h with the indicated concentrations of L-AA. **(C,D)** HeLa cells (2 × 10^5^ cells/well) were incubated for 24 h with the indicated concentrations of **(C)** sodium ascorbate or **(D)** indicated reagents. Cell lysates were subjected to Western blot analysis using antibodies against Nrf2, p62, e-IF2α, and p-e-IF2α. ACTN was the protein loading control. The results are representative of three independent experiments. Protein bands were quantified through pixel density scanning and evaluated using ImageJ, version 1.44a (http://imagej.nih.gov/ij/). The fold was normalized to the internal control protein (ACTN).

The primary etiology of human cervical cancer is human papillomavirus (HPV) infection. In order to explore the role of HPV in the actions of pharmacological concentrations of L-AA in cervical cancer cells, we compared HeLa cells, which are HPV 18-positive, with two other human cervical cancer cell lines: HPV-negative C33A cells and HPV 16-positive SiHa cells (Figures 6A,B) (31). As in HeLa cells, L-AA consistently decreased levels of Nrf2 and p62 while increasing the p-eIF2α/eIF2α ratio in both C33A and SiHa cells. This suggests that HPV may not be related to the effects of pharmacological concentrations of L-AA in cervical cancer cells.

We also tested the possibility whether the weak acidity of L-AA was responsible for its observed effects by assessing the effects of sodium ascorbate salt and acetic acid in HeLa cells. We observed that the effects of sodium ascorbate on protein levels of Nrf2, p62, and p-eIF2α are similar to those of L-AA (Figure 6C), and acetic acid or DMSO solvent for L-AA exerted no further effect (Figure 6D). Therefore, we can rule out the potential effects of weak acidity and DMSO in the actions of pharmacological concentrations of L-AA in cervical cancer cells.

### L-AA Decreases the Oxygen Consumption Rates (OCR) and Extracellular Acidification Rate (ECAR) in HeLa Cells

**Figure 7:**
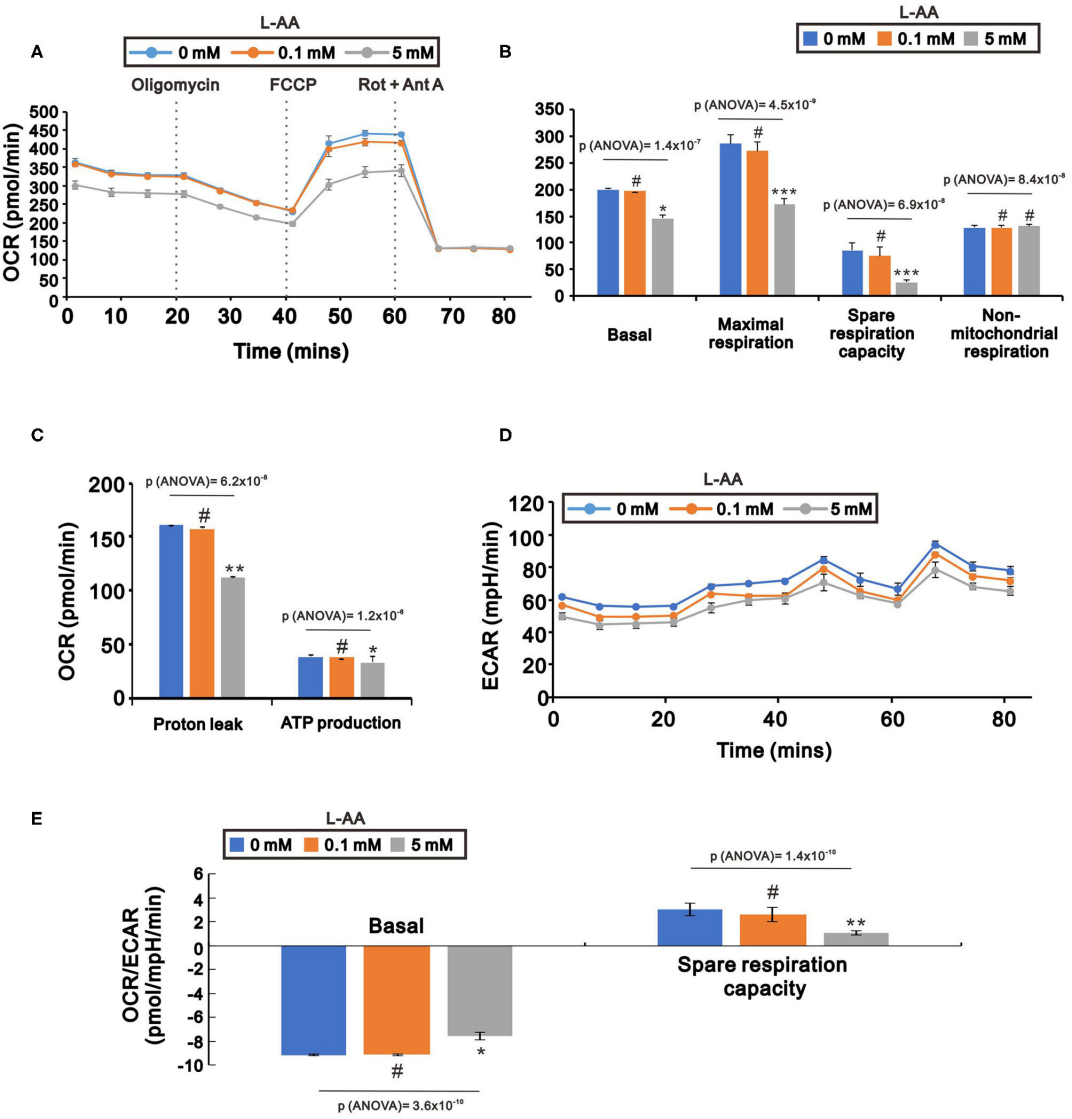
Effects of L-AA on oxygen respiration and glycolysis in HeLa cells. HeLa cells (5 × 10^3^ cells/well) were incubated for 24 h with the indicated concentration of L-AA, after which the cellular OCR **(A–C)**, ECAR **(D)**, and OCR/ECAR ratio **(E)** were measured by XF24 bioenergetic assays. The results are representative of three independent experiments. ^#^*p* > 0.05, ^*^p < 0.05, ^**^p < 0.01, and ^***^p < 0.001 (Student's *t*-tests).

ROS production occurs primarily in the mitochondria. We therefore used Seahorse XF technology to examine whether pharmacological concentrations of L-AA affect mitochondrial function, including mitochondrial respiration and glycolysis. We found that the average OCR was decreased by 5 mM L-AA (Figure 7A). In addition, detailed analysis showed that basal and maximal respiration, spare respiration capacity, ATP production, and proton leak were all significantly decreased at 5 mM but not 0.1 mM L-AA (Figures 7B,C). On the other hand, there was no change of non-mitochondrial respiration. The ECAR gradually decreased with increasing concentrations of L-AA (Figure 7D), and the OCR/ECAR ratio was significantly suppressed by 5 mM L-AA in the context of basal respiration and spare respiration capacity (Figure 7E).

### The Synergistic Effects of L-AA Plus Cisplatin or Doxorubicin on Antitumor Activity

**Figure 8:**
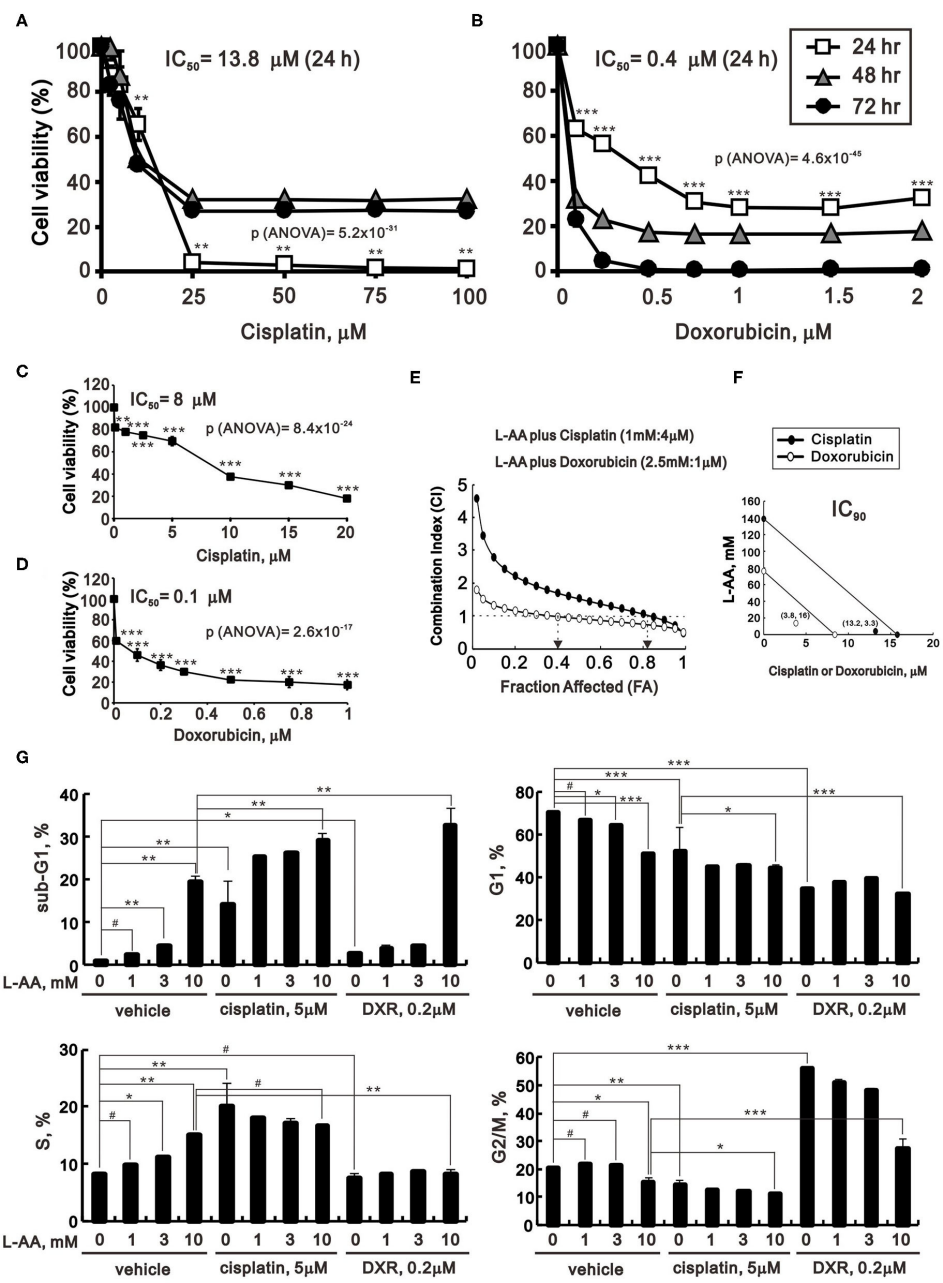
Effects of L-AA in combination with cisplatin or doxorubicin in HeLa cells. **(A,B)** HeLa cells (2 × 10^5^ cells/well) were treated with the indicated concentration of cisplatin **(A)** or doxorubicin **(B)** for 24, 48, or 72 h. **(C,D)** HeLa cells (2 × 10^5^ cells/well) were incubated with the indicated concentration of cisplatin plus 2 mM L-AA for 24 h **(C)** or with doxorubicin plus 2.5 mM L-AA for 20 h **(D)**. Cell viability was measured using the MTT method. The results are representative of three independent experiments. **(E,F)** HeLa cells (2 × 10^5^ cells/well) were incubated with designed concentration combination of L-AA, Cisplatin, or Doxorubicin for 24 h. Cell viability was measured by the MTT method, after which **(E)** the combination index of L-AA plus Cisplatin (1 mM:4 μM) (closed circle) or L-AA plus Doxorubicin (2.5 mM:1 μM) (open circle) (CI) and **(F)** IC_90_ of Cisplatin (closed circle) or Doxorubicin (open circle) were calculated using CalcuSyn (Biosoft, Cambridge, UK). **(G)** HeLa cells were incubated with the indicated concentration of L-AA plus 5 μM cisplatin or 0.2 μM doxorubicin for 24 h. The cells were then subjected to flow cytometric cell cycle profile analysis. The results are representative of three independent experiments. ^#^*p* > 0.05, ^*^p < 0.05, ^**^p < 0.01, and ^***^p < 0.001 (Student's *t*-tests).

Our present findings suggest that pharmacological concentrations of L-AA may induce ROS generation within cancer cells. We next tested the effects of combining a pharmacological concentration of L-AA with cisplatin or doxorubicin, two anticancer agents, for 24-, 48-, and 72-h treatments in HeLa cells. Administered individually, the IC_50_ values for 24-h treatments with cisplatin or doxorubicin were about 13.8 and 0.4 μM, respectively (Figures 8A,B). When administered in combination with 2 mM or 2.5 mM L-AA, however, the IC_50_ values for cisplatin and doxorubicin were significantly decreased to 8 and 0.1 μM, respectively (Figures 8C,D).

Based on these IC_50_ values and classical experimental design (25), we designed these concentration combinations and calculated the combination index (CI) between L-AA and cisplatin or L-AA and doxorubicin in HeLa cells. The CI <1, as a synergistic effect, was observed at the combination of L-AA plus Cisplatin (1 mM:4 μM) and L-AA plus Doxorubicin (2.5 mM:1 μM) in HeLa cells (Figure 8E). The fraction affected (FA) values were around 0.8 for cisplatin and 0.4 for doxorubicin (Figure 8E). We further analyzed the IC_90_ of two combinations from the data calculated for the CI. The therapeutic concentrations of cisplatin were decreased from 15.8 to 13.2 μM (at 3.3 mM L-AA), and those of doxorubicin were decreased from 8.4 to 3.8 μM (at 16 mM L-AA) (Figure 8F). This finding suggests that the benefit of clinical application of doxorubicin plus L-AA is better than cisplatin plus L-AA in human cervical cancer treatment.

The effects of cisplatin and doxorubicin on the cell cycle profile of HeLa cells were analyzed using flow cytometry (Figure 8G). By itself, cisplatin induced a subG1 population with decreases in the G1, S, and G2/M populations, while doxorubicin alone increased the G2/M population and decreased the G1 and S populations. Addition of L-AA plus cisplatin dose-dependently induced the subG1 population and further decreased the G1, S, and G2/M populations, while addition of L-AA plus doxorubicin increased the subG1 population and decreased the G2/M population.

## Discussion

**Figure 9:**
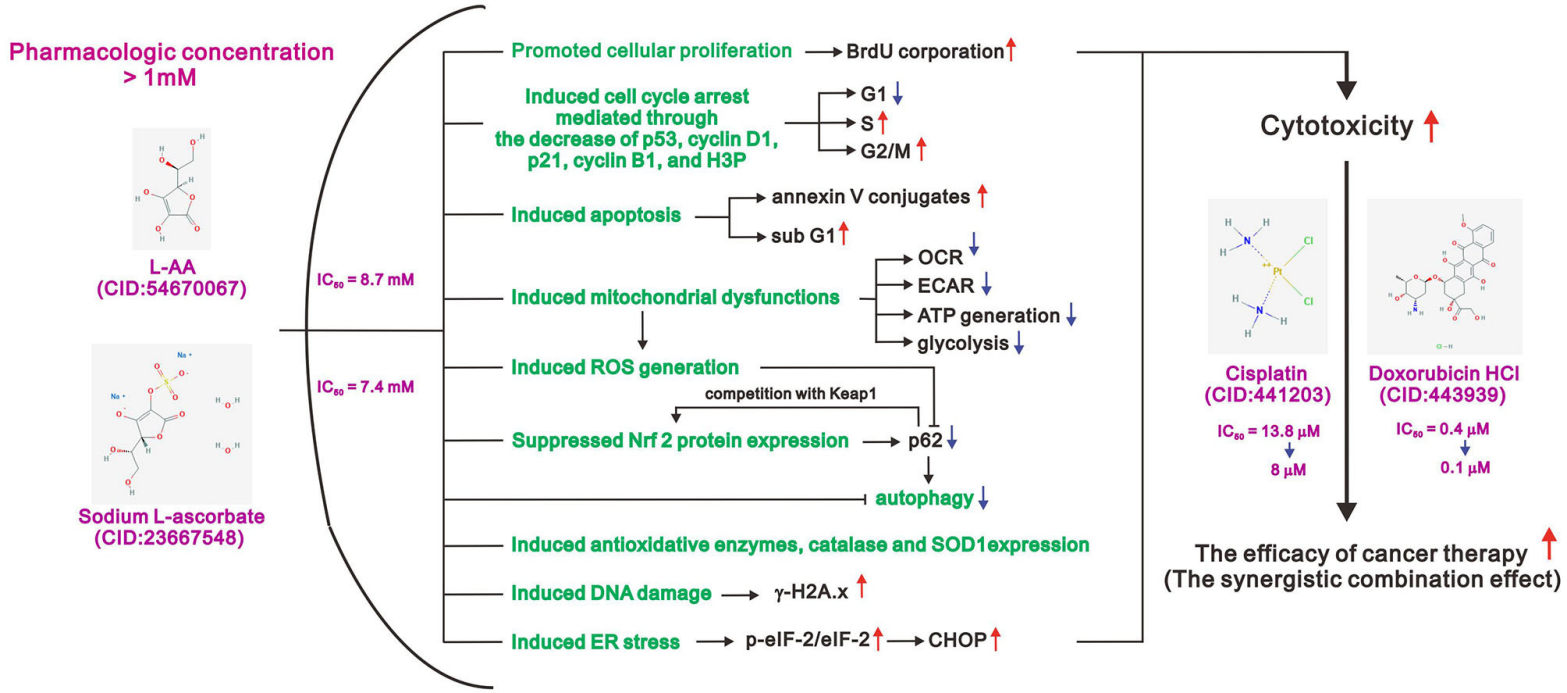
The working model of pharmacologic L-ascorbates (L-AA or sodium ascorbate) in human cervical carcinoma cells.

Given the potential safety and benefits of intravenous L-AA as a new treatment for cancer, we sought to understand the mechanism by which it acted. We found that pharmacological, but not physiological, concentrations of L-AA increased ROS levels within human cervical cancer cells. In addition, pharmacological concentrations of L-AA decreased levels of several cell cycle-related proteins, including p53, p21, cyclin D1, and H3P; induced DNA damage, as indicated by increases in γH2A.x; decreased levels of the anti-oxidative transcription factor Nrf2; and increased levels of the antioxidant enzymes catalase and SOD1 as well as two indicators of ER stress, p-eIF2α/eIF2α ratio and CHOP. L-AA may also promote cell proliferation and induce apoptosis and mitochondrial dysfunction. Finally, we demonstrated the synergistic effects of a pharmacological concentration of L-AA in combination with two first-line chemotherapeutic drugs, cisplatin, and doxorubicin. These findings provide insight into the potential cancer therapeutic efficacy of pharmacological concentrations of L-AA (Figure 9).

ROS-induced DNA damage at high-dose L-AA was observed in gene-mutated cancer cells (32). In our study, L-AA increased the levels of DNA damage marker γH2A.x, affected cell cycle profile, and decreased the levels of cell cycle-related proteins, including cyclin B1, cyclin D1, H3P, and p21. The increased S population might be attributed to S phase arrest or prolonged cellular proliferation, which was verified by BrdU assay. In addition, G2/M phase arrest had also been observed with longer incubation time of L-AA (Figure 2A for 40 h and Figure 8E for 24 h). It is generally accepted that DNA damage could induce apoptosis, and both of them are the primary cause behind the cytotoxic effects of DNA-binding antitumor drugs, for example, cisplatin and doxorubicin. The combination of L-AA with cisplatin or doxorubicin significantly elevated the subG1 population, along with evident decreases in the G1 and G2/M populations in current work. Moreover, consistent with previous studies (33, 34), cisplatin could cause S phase arrest, while doxorubicin led to G2/M phase arrest (Figure 8E). More importantly, when combined with L-AA, the IC_50_ values of cisplatin and doxorubicin could be reduced from 13.8 to 8 μM and from 0.4 to 0.1 μM, respectively. On the other hand, ROS could also be involved in the apoptotic processes, but the precise mechanisms remain to be investigated in the future.

The possibility of exploiting elevated oxidative stress, achieved through inhibition of antioxidant enzyme activity or production of ROS within cancer cells, as a potential anticancer treatment strategy was examined and reported in many studies (16–18, 35, 36). Oxidation of L-AA produces hydrogen peroxide, which can initiate Fenton's reactions and cause oxidative damage to cellular macromolecules (2, 37, 38). Moreover, recycling of endogenous L-AA enables continuous production of hydrogen peroxide (39), while intravenous injection of L-AA, independent of its intracellular levels, will effectively increase its extracellular concentrations. However, the precise equilibria among intracellular and extracellular L-AA, hydrogen peroxide, and the formation of hydroxyl radicals through Fenton's reaction remain to be investigated in the future.

Nrf2 is a critical transcription factor mediating the expression of intracellular antioxidants, such as catalase and SODs, and phase II detoxification enzymes in normal cells. Therefore, the accumulation of Nrf2 in cancer cells is beneficial to cell proliferation and protects cancer cells from chemotherapeutic agents, oxidative stress, and radiotherapy, resulting in a poor prognosis (40, 41). Here, L-AA reduced the IC_50_ values of cisplatin and doxorubicin and levels of Nrf2 protein through effects at the transcriptional, translational, and post-translational stages. However, some Nrf2-depednet antioxidant proteins, such as catalase and SOD1, were increased. These results seemed inconsistent with the idea that L-AA might render cancer cells more susceptible to chemotherapeutic agents by inhibiting the Nrf2-dependent protective response. The possible reasons behind are that the catalase could be upregulated by ER stress-related splicing Xbp-1 in HeLa cells, and numerous SOD1 post-translational modifications have been implicated in maintaining SOD1 stability, suggesting that Nrf2 is just one of transcription factors responsible for catalase and SOD1 expressions (42, 43). In summary, the effects of L-AA might be determined by the balance between the antioxidant capability and ROS induction, as regulated by Nrf2, while the increased protein levels of catalase and SOD1 by L-AA might be a consequence of elevated ROS generation. However, the antioxidative capability affected by L-AA remains to be quantified in the future. Besides, the protein levels of Nrf2 and p62, a Nrf2 downstream target, were decreased by L-AA. It remains unclear whether decreases in p62 levels ameliorated the competition with Keap1, leading to Nrf2 destabilization, or the reduction in Nrf2 expression led to much weaker *p62* transcription in the presence of elevated ROS levels.

Redoxome and bioinformatics analyses in L-AA-treated breast cancer cells are also consistent with our present findings that L-AA affects cell proliferation, mRNA translation, and eIF2α signaling (44, 45). Moreover, L-AA appears to induce mitochondrial dysfunction leading to decreases in ATP production and glycolysis. The difference in energy metabolism between cancer and normal cells, known as the Warburg effect, likely renders cancer cells far more vulnerable to glycolysis impairment by L-AA than their normal counterparts. However, other factors, including tumor environment (or density), rapid ATP synthesis, protein biosynthesis, and cell signaling via ROS should be addressed to clarify the mechanism of action of L-AA in cancer cells. We found that L-AA exhibited similar effects in three human cervical cancer cell lines, Hela cells, C33A cells, and SiHa cells. Since C33A is a non-HPV infected cell line and expresses p53R273C mutation, it suggests that HPV and p53 might not contribute to our current findings of the cytotoxicity of L-AA in human cervical cancers (31).

Kuiper et al. reported that, *in vivo*, high HIF-1α activation and tumor growth were related to low tumor tissue ascorbate levels (46). In the present study, various concentrations of L-AA had no apparent effect on HIF-1α levels (data not shown). That L-AA functions as an enzyme cofactor for Fe^2+^-2-oxoglutarate-dependent dioxygenases suggests that fine tuning of the intracellular ascorbate levels may limit the expression of HIF-1α and angiogenic proteins, which promote tumor growth and poor outcome. In addition, intravenously administered ascorbate also appears to affect DNA demethylation through the Tet2 enzyme, another Fe^2+^-2-oxoglutarate-dependent dioxygenase (7, 47). Under homeostatic conditions, L-AA regulates the balance between self-renewal, differentiation, and cell death of hematopoietic stem cells and pluripotent progenitors by promoting Tet2 activity (4, 10, 47). Thus, L-AA may serve as an ROS modulator, a hypoxia stimulator, an epigenetic editor, and an enzyme cofactor during cancer therapy.

In conclusion, at pharmacological concentrations, L-AA not only induced ROS production and DNA damage but also decreased the levels of several cell cycle-related proteins and the transcription factor Nrf2 and increased the levels of catalase, SOD1, and ER stress-related proteins. L-AA may also promote cell proliferation and induce apoptosis and mitochondrial dysfunction through the elevation of ROS. In addition, pharmacological concentrations of L-AA appeared to increase the susceptibility of HeLa cells to cisplatin and doxorubicin. These findings provide insight into how adjustment of the cellular ROS status through L-ascorbate (L-AA or sodium ascorbate) administration could potentially enhance the efficacy of cancer therapies.

## Data Availability Statement

The original contributions presented in the study are included in the article/supplementary material, further inquiries can be directed to the corresponding author/s.

## Author Contributions

T-MW and S-TL carried out experiments, analyzed data, and wrote the paper. S-YC analyzed data and wrote the paper. G-SC, C-CW, and S-MH conceived of the study, participated in its design and coordination, and helped to draft the manuscript. All authors contributed to the article and approved the submitted version.

## Conflict of Interest

The authors declare that the research was conducted in the absence of any commercial or financial relationships that could be construed as a potential conflict of interest.

## Abbreviations

7-AAD: 7-Amino-Actinomycin
ACTN: α-actinin
L-AA: L-ascorbic acid
BrdU: 5-bromo-2-deoxyuridine
CHX: cycloheximide
CI: combination index
DCFH-DA: 2',7-dichlorofluorescein diacetate
DMEM: Dulbecco's modified Eagle's medium
ER: endoplasmic reticulum
FA: Fraction affected
FACS: fluorescence- activated cell sorting
FBS: fetal bovine serum
FCCP: carbonyl cyanide 4-[trifluoromethoxy] phenylhydrazone
FITC: fluorescein isothiocyanate
H3P: phosphorylation at Ser 10 Histone H3
HPV: human papillomavirus
H_2_O_2_: hydrogen peroxide
HIF1α: hypoxia-inducible factor 1 alpha
HO-1: Heme oxygenase 1
MTT: thiazolyl blue tetrazlium bromide
NAC: N-acetyl cysteine
OCR: oxygen consumption rate
PBS: phosphate buffered saline
PI: propidium iodide
ROS: reactive oxygen species
RLU: relative light units
RPMI: Roswell Park Memorial Institute
RT-PCR: reverse transcription-polymerase chain reaction
SOD1: superoxide dismutase 1
Tet2: ten-eleven translocation 2.
